# Protective effect of hydrogen-rich saline on pressure overload-induced cardiac hypertrophyin rats: possible role of JAK-STAT signaling

**DOI:** 10.1186/s12872-018-0773-9

**Published:** 2018-02-13

**Authors:** Zhixin Fan, Yufei Gao, Zhiwei Huang, Fenghua Xue, Shujing Wu, Jing Yang, Liqun Zhu, Lu Fu

**Affiliations:** 1 Department of Cardiovascular Medicine, First Affiliated Hospital of Harbin Medical University, 23 Youzheng Street, Nangang District, Harbin, Heilongjiang 150001 China; 2 Emergency Department, Heilongjiang Provincial Hospital, Harbin, China

**Keywords:** HRS, Cardiac hypertrophy, Signaling pathway

## Abstract

**Background:**

Molecular hydrogen has been shown to have antioxidant effect and have been used to prevent oxidative stress-related diseases. The goal of this study was to explore if hydrogen-rich saline (HRS) plays a cardioprotective effect on abdominal aortic constriction (AAC) induced cardiac hypertrophy in rats. 60adult Sprague–Dawley rats received surgically the AAC for 6-week. After the surgery, the rats were randomly divided into 4 groups (15 for each):1: sham-operated (sham); 2: AAC-model; 3: AAC + Low HRS (LHRS); and 4: AAC + High HRS (HHRS). The rats in sham and AAC-model groups were treated with normal saline intraperitoneally, while rats in LHRS and HHRS groups were intraperitoneally treated with 3 or 6 mL/kg HRS daily, respectively, for 6-week.

**Results:**

The ratios of HW/BW and LVW/BW were shown in an order of Model > LHRS > HHRS > SHAM groups. The cardiac hypertrophy was also manifested with increased expressions of atrial natriuretic peptide (ANP), brain natriuretic peptides (BNP) and fibrosis of cardiac tissues in AAC-model group, which could likewise be restrained in LHRS and HHRS groups. Moreover, the JAK-STAT (Janus Kinase-Signal transducers and activators of transcription) signaling molecule expressions were decreased with HRS treatment.

**Conclusions:**

Our results showed a protective effect of HRS on pressure overload-induced cardiac hypertrophy in rats, which may be associated to a decreasing in JAK-STAT signaling pathway.

## Background

Cardiac hypertrophy is characterized by myocardial cell enlargement which involves physiological and pathological hypertrophy. End stage of pathological cardiac hypertrophy leads to heart failure and is associated with high mortality. Pathological cardiac hypertrophy contains the interstitial and perivascular fibrosis, apoptosis and the increased ANP (atrial natriuretic peptide) and BNP (brain natriuretic peptide) synthesis. Hydrogen (H_2_) has been assayed in experimental settings in humans [1] to exert the protective effects against tissue damages including brain ischemia, neonatal brain hypoxia-ischemia, liver lesions, lung lesions, and myocardial ischemia/reperfusion injury through antioxidant activity [2–6]. Furthermore, HRS shows an anti-inflammatory effect [7], as reported that hydrogen-containing saline therapy ameliorated doxorubicin-induced cardiac hypertrophy and heart failure in rats [8]. The Janus kinase/signal transducer and activator of transcription (JAK/STAT) pathway triggers multiple signals involved in development, homeostasis and inflammation. The JAK-STAT signaling also plays a central role in transducing stress and growth signals in the hypertrophic heart. Ligand binding to receptors activates JAK and STAT proteins which are phosphorylated and form homo or heterodimers which then translocate into the nucleus where they regulate gene expression. In mammals, the JAK/STAT pathway transduces signals for a wide array of cytokines and growth factors including AngII, TNF-α, IL-1β, IL-6 and IFN-γ, all of which have been involved in cardiac hypertrophy.

Based on the previous study, we established a pressure overload-induced cardiac hypertrophy model in rats. We hypothesized that HRS prevents the cardiac hypertrophy in rats, which might be associated with decreased JAK-STAT signaling pathway. The anti-hypertrophic effect of HRS might restrain the progression of cardiac hypertrophy.

## Methods

All the experiments were approved by the Animal Care Committee of Harbin Medical University (Heilongjiang, China).

### HRS production

Hydrogen-rich saline was prepared as previous description [9]. Briefly, H_2_ was dissolved in 0.9% saline for 6 h under high pressure (0.4 MPa) to a supersaturated level using a HRS-producing apparatus. The saturated HRS was stored at 4 °C in an aluminum bag without dead volume and sterilized by gamma radiation. To confirm the concentration of hydrogen in the saline, gas chromatography was performed. HRS was freshly prepared every week to ensure a concentration of more than 0.6 mmol/L.

### Cardiac hypertrophy model and animal grouping

Male Sprague–Dawley rats, weight 200–220 g, were provided from the Experimental Animal Center of The First Affiliated Hospital of Harbin Medical University (Harbin, China). Rats were housed with free access to food and water under a natural day/night cycle. Rats were acclimated for 7 days before any experimental procedures. All rats were cared according to Laboratory Animal Administration Rules (China, 2013).

Sixty rats were randomly divided into 4 groups: 1:sham-operated (sham); 2: AAC-model;3:AAC + Low HRS of 3 ml/kg(LHRS); and 4:AAC + High HRS of 6 ml/kg (HHRS) groups with 15 rats for each group. HRS was injected into the peritoneal cavityonce a day after surgery. The model of cardiac hypertrophy was established with the constriction of abdominal aorta as described previously [10]. Briefly, after the rat was anesthetized, and placed in supine position, a 2-cm incision along the midline of the abdomen was made. After the abdominal aorta was identified, an 8-cm length of 4–0 silk suture was passed underneath the abdominal aorta between the origins of the right and left renal arteries, and a knot was made around the aorta and a 22G needle, and then the needle was removed immediately to achieve a 0.7-mm diameter constriction. Sham-operated rats received a same surgical procedures without constriction performed.

At 6-week after AAC and HRS treatments, rats were weighed and then killed with a quick decollation. The hearts were soon removed, and rinsed in ice-cold PBS. The body weight, heart weight and the left ventricular weight were determined.

### Histological analysis

The left ventricles were dissected and immersed in 4% paraformaldehyde overnight. Then the left ventricles were paraffin-embedded, and sectioned at a thickness of 4 μm. Masson staining were performed for light microscopy. The collagen volume fraction (CVF) was calculated as collagen area /total tissue area in a field.

### Western blot analysis

The left ventricles were harvested and Western blot analysis was carried out as described previously [11], total protein concentration was determined by Bradford reagent (Bio-Rad), and the expression of atrial natriuretic peptide (ANP) and brain natriuretic peptide (BNP) in left ventricles were determined with western blot. Briefly, about 50 μg of sample was separated by electrophoresis on a 4–12% SDS-polyacrylamide NuPAGE gradient gel (Invitrogen) and transferred to a PVDF membrane followed by probing with primary antibodies and followed by secondary antibodies. The protein bands were visualized using chemiluminescent substrates (Pierce). ANP and BNP antibodies (1/1000, respectively) were from Millipore (Temecula, CA) and antibodies of IL-6,JAK, STAT3 and p-STAT3were from Abcam (Cambridge, MA). Densitometry analysis of the bands was conducted with Scion Image Beta 4.02 software. The quantitative measurements of IL-6, JAK and STAT3 expression in the heart tissue were assessed with commercial ELISA kits following the manufacturer’s instructions. Absorbance was read on a microplate reader, and the concentrations were determined based on its standard curve. Native proteins from the left ventricles were isolated using Total Protein Extraction Kit (Biochain Institute, Inc.).

### Apoptosis analysis

The apoptotic cells were detected byterminal deoxynucleotidyl transferase (TdT) dUTP Nick-End Labeling (TUNEL) assay (Promega) according to manufacturer’s instructions. The apoptotic index’s quantification was conducted at 400 X. In all groups, 20 fields were randomly picked and the apoptotic index of each field was estimated as the percent of TUNEL-positive cells.

### Statistics analysis

The data are expressed as mean ± S.D. Statistical analysis was performed with SPSS 11.5 (SPSS Inc., Chicago, IL, USA). Statistical comparisons were conducted through one-way analysis of variance with post hoc test of Student–Newman–Keuls. AP value of less than 0.05 was considered statistically significant.

## Results

**Fig. 1:**
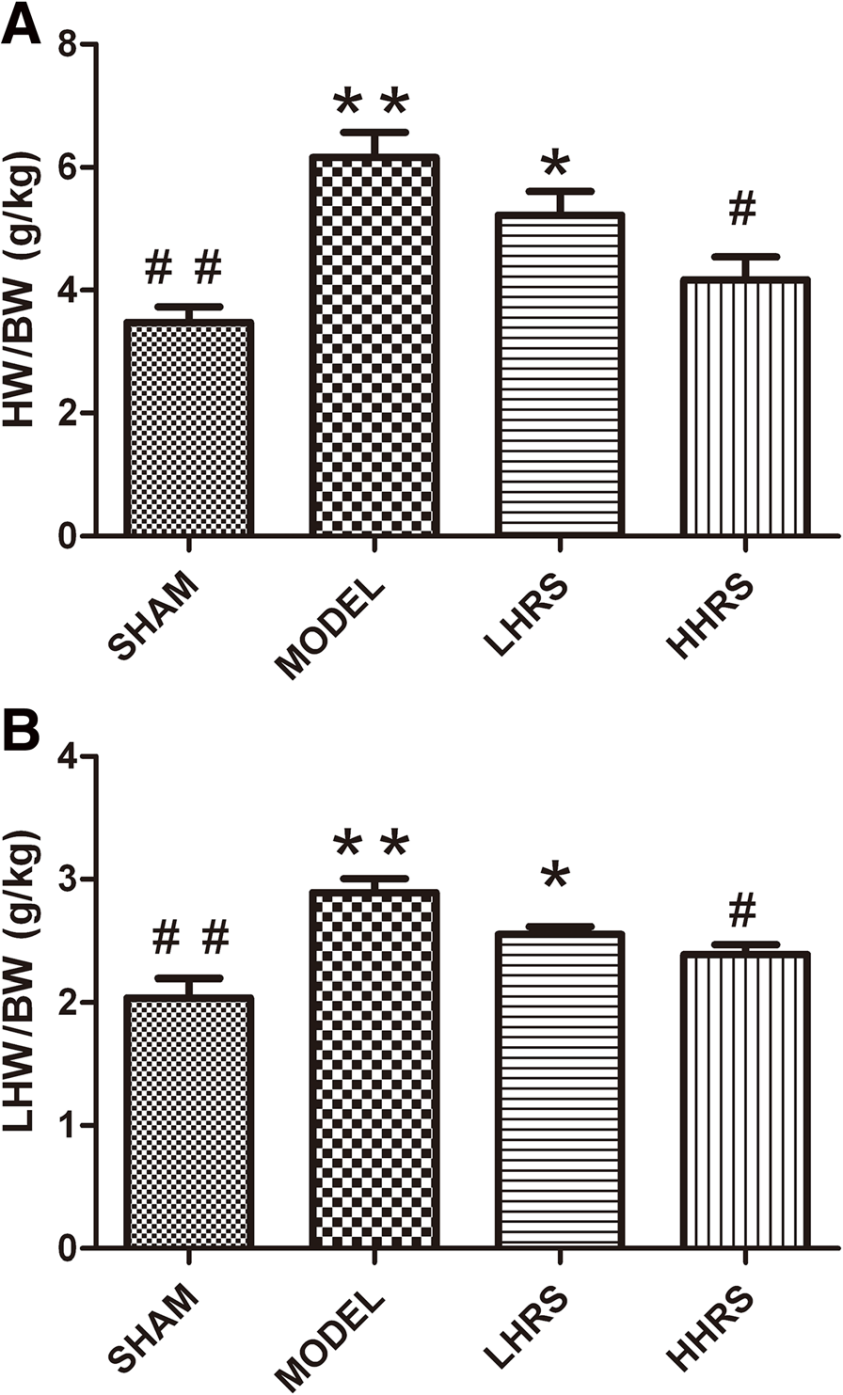
Effects of HRS on HW/BW (**a**) and LHW/BW (**b**) in each group. For all Figures, the quantitative data were expressed as mean ± SD, *n* = 15 per each group. *P<0.05, **P<0.01 vs. SHAM group; ^#^*P*<0.05, ^##^*P*<0.01 vs. AAC-model group

**Fig. 2:**
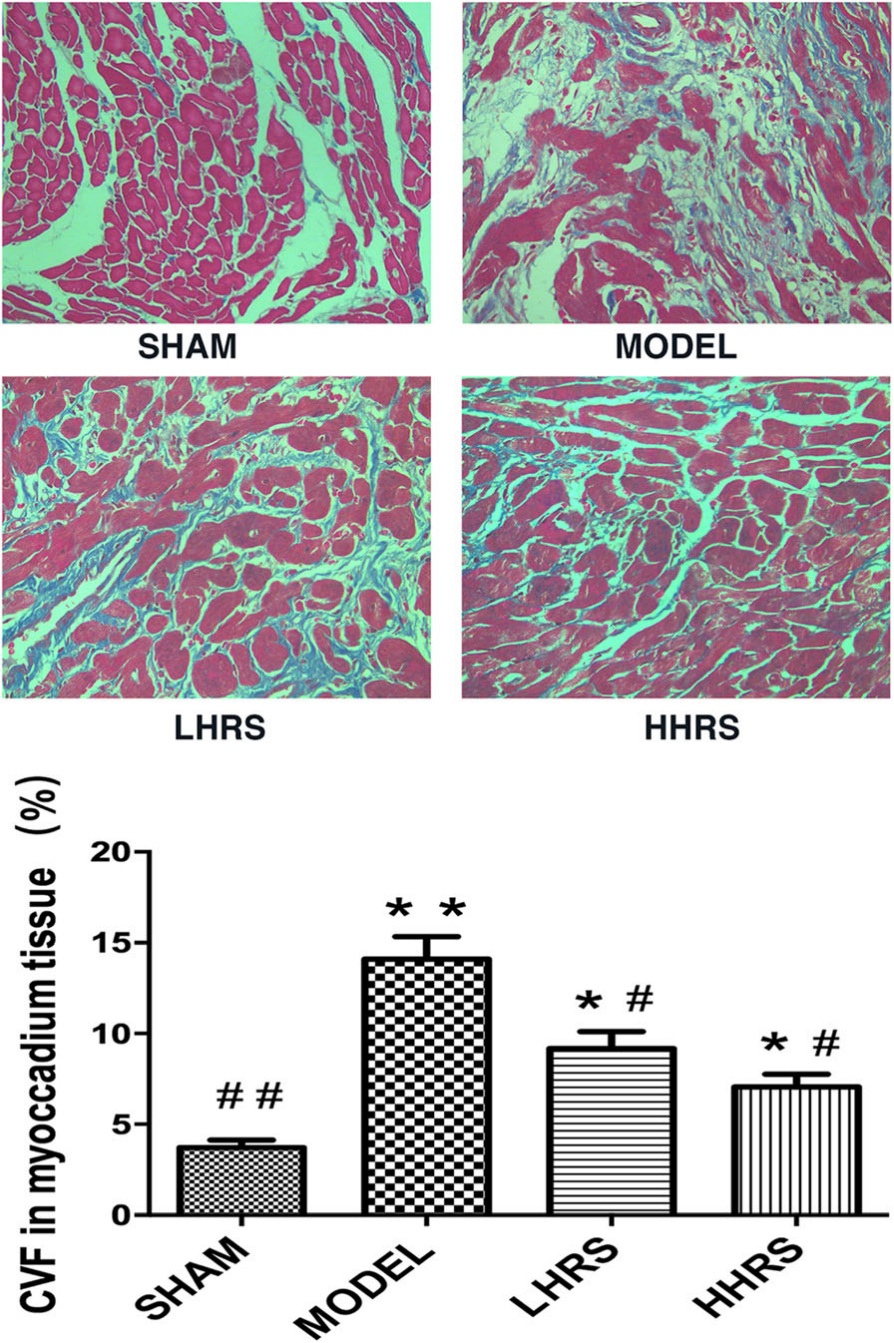
Upper panel: representative photomicrographs of left ventricle tissue with Masson staining (400× magnification). The AAC-model group showed the increased tissue interstitium and collagen protein expression (shown in blue color), while the LHRS and HHRS groups showed significantly less collagen protein expression. Low panel: quantitative calculation of collagen protein expression based on blue color volume

**Fig. 3:**
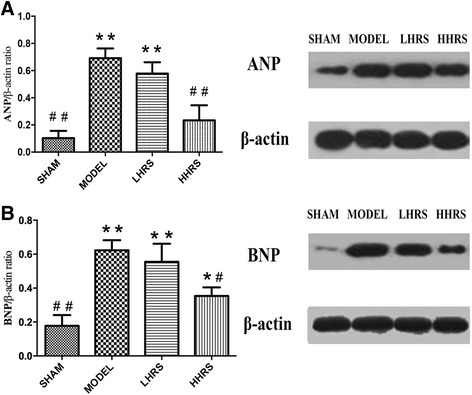
The expression of ANP (**a**) and BNP (**b**) inthe left ventricle tissue was detected with western blot. Images from western blotting were on the right. The densitometry analyses of the bands, showing the ratio of ANP/beta-actin and BNP/beta-actin, were on the left. HRS treatment with 3 or 6 ml/kg reduced the expression of hypertrophy protein markers

**Fig. 4:**
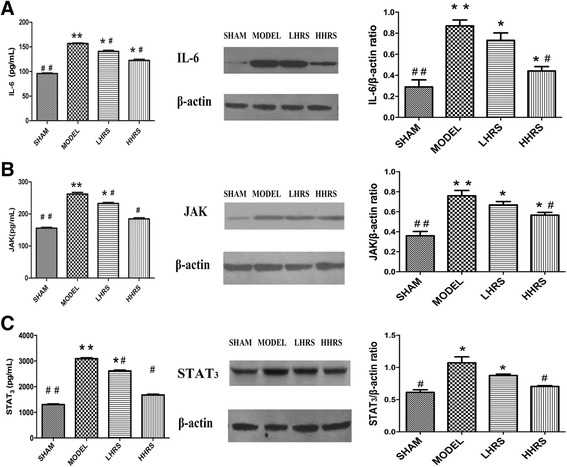
Expressions of IL-6 (**a**), JAK (**b**) and STAT3 (**c**) in left ventricle tissue. On the left columns, the absolute levels of IL-6, JAK and STAT3 were measured with Elisa. The middle columns were the representative western blot images, and the right columns were the ratios of densitometry analyses from western blot bends. HRS treatments were in 3 or 6 ml/kg

**Fig. 5:**
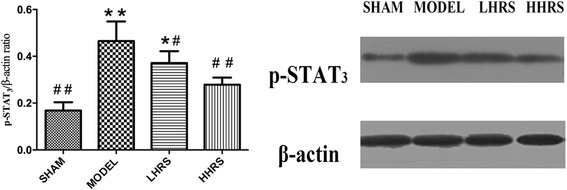
HRS treatment reduced p-STAT3 levels in the left ventricle tissue. Western blotting analyses were performed on lysates from left ventricular tissues to assay p-STAT3. Compared with the sham-operated rats, the p-STAT3 expression levels in the AAC-treated rat hearts were significantly increased, which were prevented with treatment of HRS of 3 or 6 ml/kg

Cardiac hypertrophy and remodeling plays a critical role in the development of heart failure. Hypertensive heart disease is the key contributing factor of cardiac hypertrophy. To mimic these conditions, here we use a rat model whose abdominal aorta was constricted to increase the resistance of the left ventricle, and ultimately lead to a pressure overload in the heart. In this rat model, cardiac hypertrophy and remodeling were observed 6 weeks after the abdominal aortic constriction surgery. The averages of body weight, heart weight and left ventricle weight from each group were listed in Table 1. There were the increased ratios of HW/BW and LVW/BW in an order of model > LHRS>HHRS > SHAM groups (Fig. 1). The two different doses of HRS treatment for 6 weeks could prevent the cardiac hypertrophy, which was characterized with decreased ratios of HW/BW (Fig. 1a) and LVW/BW (Fig. 1b). The histological images of the hearts from the AAC-model group rats showed that the degrees of myocardial and perivascular fibrosis at the cross-section was significantly increased compared with the sham-operated rats, and the HRS-treated rats (Fig. 2). The levels of BNP, ANP (Fig. 3), JAK and IL-6 (Fig. 4) in the left ventricle tissue were markedly reduced in two-dose of HRS treated groups compared with AAC-model group. However, the levels of STAT3 were almost same among 4 groups, but p-STAT3 protein level was the highest in the AAC-model group, which was reduced with HRS treatment (Fig. 5).

**Table 1:** Comparison of body weight, heart mass weight and left ventricular weight

	n	BW (g)	HW (mg) LVW (mg)
SHAM	15	309.15 ± 11.05	912 ± 34.38^##^626.57 ± 11.51^##^
MODEL	15	285.16 ± 8.04	1579.46 ± 65.38**835.13 ± 44.79**
HHRS	15	292.25 ± 5.451341.23 ± 58.72*	742.96 ± 17.59*
LHRS	15	296.75 ± 6.021130.22 ± 79.88^#^	701.56 ± 11.85^#^

The data were expressed as mean ± SD, *n* = 15.*P<0. 05, **P<0. 01 vs. Sham group; #*P*<0. 05, ##*P*<0. 01 vs. model group

**Fig. 6:**
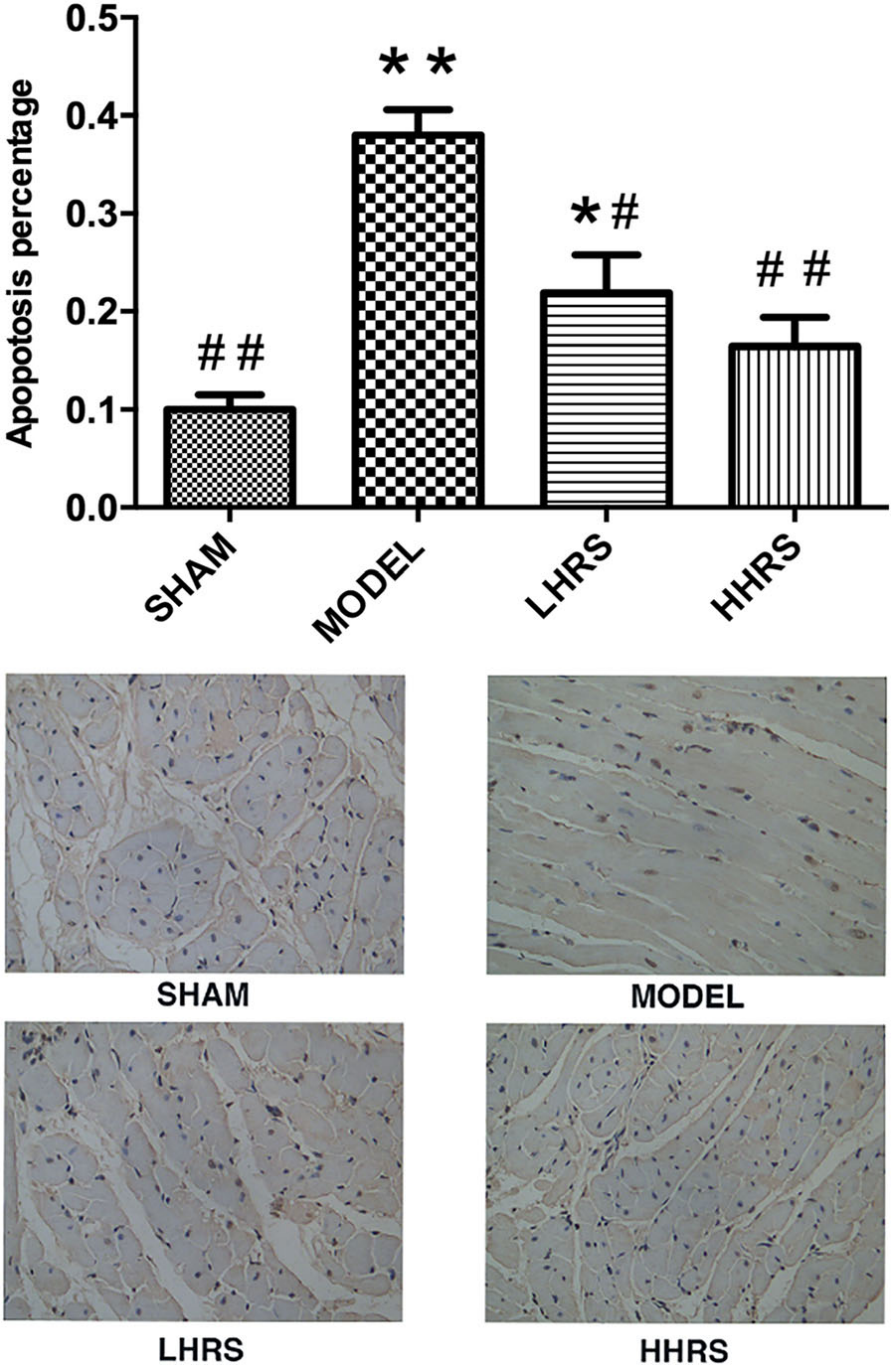
HRS protected the cardio myocytes from apoptosis. The apoptosis levels in the hearts of AAC-model rat were significantly high, and this high level was prevented with treatment of HRS in 3 or 6 ml/kg, respectively. Low panel: images from TUNEL assay; Upper panel: quantitative calculation from the images

The apoptotic analysis showed that apoptosis levels were significantly increased in the heart of AAC-model rats. However the treatment of HRS significantly decreased the apoptosis compared with AAC-model rats (Fig. 6).

## Discussion

The study showed that HRS remedy could prevent the process of cardiac hypertrophy, which is induced with cardiac pressure overload caused by AAC in a rat model. This conclusion was supported by the findings from histological and biochemistry studies. HRS reduced IL-6 levels and modulated cardiac cell activity in heart tissue. Furthermore, HRS reduced JAK-STAT signaling pathway, which has been shown to associate with heart hypertrophy.

In this model, the abdominal aorta is constricted above the renal arteries in rat to increase cardiac pressure overload and induce cardiac remodeling, hypertrophy and finally heart failure [12]. Decompensated cardiac remodeling were characterized by pulmonary congestion and right ventricular hypertrophy 6 weeks after AAB [13, 14]. This AAC rat model was characterized by using LVH and LV working disorders, along with hypertension.

In this study, we showed cardiac tissues remodeling which responds to pressure overload induced by constriction the abdominal aorta; these symptoms were reversed by HRS treatment. These outcomes indicate that HRS can reverse cardiac remodeling.

Hypertensive disease is the most frequent background of LVH, and anti-hypertensive remedy should not only reduce blood pressure but also lead to the regression of LVH [15, 16]. HRS decreasing cardiac hypertrophy in our study suggests that HRS can prevent against hypertensive cardiovascular events. Previous studies have concentrated on HRS’ effects on organ injury caused by ischemia/reperfusion; HRS can decrease apoptosis and inflammation in various kinds of diseases [17, 18]. However, the effect of HRS on cardiac hypertrophy stays unclear.

The JAK-STAT signaling pathway could increase the expression levels of genes which are related to cardiac hypertrophy [19, 20]. We hypothesized that the protective effects of HRS in the progression of pressure overload-induced cardiac hypertrophy might be in partially by lowering the JAK-STAT signaling pathway. In our animal model of cardiac hypertrophy, the activity and the expression level of several signal proteins were increased, including IL-6, JAK and p-STAT3. IL-6 was produced by neutrophils and activated macrophages and activates the inflammatory reaction. Gp130 is the common signal transducer used by the IL-6. The initiated intracellular signaling is strictly dependent on the function of gp130. Formation of gp130-containing complexes results in activation of intracellular JAKs. The phosphorylation of gp130 leads to dimerization of gp130 and activation of STAT1 and STAT3 as well as the JAK-STAT pathway. The activity of JAK-STAT signaling pathway was upregulated in the model group, which was downregulated by the treatment of HRS. In addition, the tissue IL-6 levelwas in accordance with the activity of the JAK-STAT signaling pathway. These results support our hypothesis.

## Conclusion

In conclusion, this study demonstrated that HRS treatment can prevent cardiac hypertrophy and restrained the development of cardiac hypertrophy induced by pressure overload in rats, which may be associated to a decreasing in JAK-STAT signaling pathway.

## Abbreviations

AAC: Abdominal aortic constriction
ANP: Atrial natriuretic peptide
BNP: Brain natriuretic peptides
HHRS: High hydrogen-rich saline
HRS: Hydrogen-rich saline
LHRS: Low hydrogen-rich saline
LVH: Left ventricular hypertrophy
